# Triglyceride Glucose Index Was a Predictor of 6-Month Readmission Caused by Pulmonary Infection of Heart Failure Patients

**DOI:** 10.1155/2022/1131696

**Published:** 2022-10-19

**Authors:** Licheng Shi, Jianan Liu, Xuanfeng Zhu, Tiantian Li, Jingli Wen, Xinyu Wang, Xu Qi

**Affiliations:** ^1^Department of Respiratory and Critical Care Medicine, First Affiliated Hospital of Nanjing Medical University, 210029 Nanjing, China; ^2^Department of Respiratory Medicine, Jiangsu Province Official Hospital, 212004 Nanjing, China; ^3^Department of Respiratory and Critical Care Medicine, XuZhou Central Hospital, 221009 Xuzhou, China

## Abstract

**Objectives:**

Insulin resistance is associated with the prognosis of heart failure (HF) patients. The triglyceride glucose (TyG) index is a simple marker of insulin resistance. However, it remains unclear whether the TyG index is associated with the incidence of readmission in patients with HF.

**Methods:**

We enrolled 901 patients with completed records on serum triglyceride and glucose in our study. The TyG index was calculated as log (fasting triglycerides (mg/dL) *x* fasting glucose (mg/dL)/2). There were 310 cases of readmission and the average TyG index was 7.8 ± 0.7. Restricted cubic spline was fitted to explore the linearity of TyG index associating with 6-month readmission of HF patients. Logistic regression analysis was performed to explore the association between TyG index quartile and the incidence of 6-month readmission.

**Results:**

Only the 6-month readmission was significantly different among TyG quartiles, and it was the highest (41.9%) in the lowest quartile (ranging 6.17∼7.36). the TyG index was nonlinearly associated with 6-month readmission (*p* for nonlinearity = 0.009), with the lower level of TyG index increasing the risk of 6-month readmission. Besides, multivariable logistic analysis showed that the lowest TyG quartile was associated with a higher incidence of 6-month readmission in the unadjusted model (odds ratio [OR] 1.74, 95% confidence interval [CI] 1.18–2.57; *p*=0.005), partially adjusted model (OR 1.82, 95%CI 1.22–2.72; *p*=0.004), and fully-adjusted model (OR 1.65, 95%CI 1.09–2.45; *p*=0.024). The association was consistent across gender and diabetes group.

**Conclusion:**

A lower TyG index independently increased the risk of 6-month readmission in HF patients, which could be a prognostic factor in heart failure.

## 1. Introduction

Heart failure (HF) remains a leading cause of death worldwide and has a poor prognosis [1]. Nearly, one quarter of HF patients are readmitted within 30 days and half are readmitted within 6 months [2, 3]. Therefore, it is of great value to identify the predictors of readmission and decrease the incidence of readmission of HF patients.

Insulin resistance is proven to be associated with the incidence of heart failure [4] and long-term cardiovascular events [5]. Recently, the triglyceride glucose (TyG) index was confirmed to be a surrogate marker of insulin resistance [6]. Previous studies demonstrated that TyG index was of prognostic value in patients with hypertension [7], coronary artery disease [8], or heart failure [9]. In addition, TyG was a novel biomarker of myocardial fibrosis in HF patients [10]. However, it has been not clear whether the TyG index was associated with the risk of readmission of HF patient.

## 2. Methods

### 2.1. Study Population

A retrospective single-center observational study was performed by integrating electronic healthcare records and follow-up outcome data, which collected information for a total of 2,008 patients with heart failure. The study was conducted at Zigong Fourth People's Hospital, Sichuan, China, from December 2016 and was approved by the ethics committee of Zigong Fourth People's Hospital (Approval Number: 2020–010). Informed consent was waived due to the retrospective design of the study [11]. Heart failure (HF) was defined according to the European Society of Cardiology (ESC) criteria [12]. After excluding HF patients without records on glucose and triglycerides, a total of 901 patients were included in our primary analysis.

### 2.2. Clinical Variables and Outcomes

The demographic data, including age, gender, and body mass index (BMI), were acquired from electronic healthcare records. Baseline examination was performed for systolic blood pressure (sBP), diastolic blood pressure (dBP), and New York Heart Association (NYHA) cardiac function on the day of hospital admission. Echocardiography was performed to obtain left ventricular ejection fraction (LVEF) and left ventricular end diastolic diameter (LVEDD). Comorbidities included a medical history of myocardial infarction (MI), congestive heart failure (CHF), hypertension, peripheral vascular disease (PAD), chronic obstructive pulmonary disease (COPD), diabetes (DM), and chronic kidney disease (CKD).

Baseline laboratory tests were performed by standard biochemistry assays on the day of hospital admission. The laboratory variables included estimated glomerular filtration rate (eGFR), brain natriuretic peptide (BNP), high sensitivity C-reactive protein (hsCRP), albumin, cholesterol, low-density lipoprotein cholesterol (LDL), high-density lipoprotein cholesterol (HDL), triglyceride, and glucose. The TyG index was calculated as log (fasting triglycerides (mg/dL) *x* fasting glucose (mg/dL)/2). Clinical outcomes were followed up for half a year. They included death within 28 days, readmission within 28 days, death within 3 months, readmission within 3 months, death within 6 months, and readmission within 6 months.

### 2.3. Statistical Analysis

Data are presented as the mean ± standard deviation. Intergroup comparisons were performed using ANOVA for continuous variables or chi-square test for categorical variables, respectively. Dose-response analysis was performed by the logistic regression models with restricted cubic splines. Multivariable logistic regression models were established to explore the association between TyG quartile and the incidence of 6-month readmission. Model 1 was unadjusted. Model 2 was adjusted for age, gender, BMI, NYHA, MI, CHF, hypertension, PAD, COPD, DM, CKD, LVEF, and LVEDD. Model 3 was adjusted for age, gender, BMI, NYHA, MI, CHF, hypertension, PAD, COPD, DM, CKD, LVEF, LVEDD, ACEI/ARB, MRA, eGFR, hsCRP, BNP, albumin, cholesterol, and LDL. Subgroup analyses was performed to explore the interactions modifying the relationship. A *p* value < 0.05 was considered statistically significant. All statistical analyses were performed with *R* software (version 3.6).

## 3. Results

**Figure 1:**
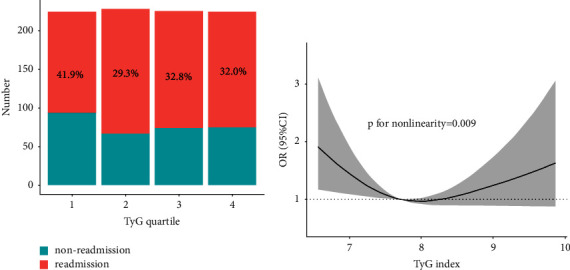
(a) The number of 6-month readmission was calculated according to TyG quartile. (b) The restricted cubic spines showed the odds ratio of readmission based on TyG index.

There were 901 patients enrolled in our study and 401 (44.5%) were male individuals. There were 310 cases of readmission and the average TyG index was 7.8 ± 0.7. The baseline clinical and laboratory characteristics of the study population are shown in Table 1. The whole population was stratified into four groups according to their TyG quartiles. The highest TyG quartile tended to have more percentage of female (*p*=0.001) and diabetes (*p* < 0.001), as well as higher levels of albumin (*p*=0.002) and cholesterol (*p* < 0.001). Besides, only the 6-month readmission was significantly different among groups, and it was the highest (41.9%) in the lowest quartile (ranging 6.17∼7.36; Figure 1(a)).

The dose-response relationship between the TyG index and the incidence of 6-month readmission was explored using the logistic regression with restricted cubic spines. As shown in Figure 1(b), the TyG index was nonlinearly associated with 6-month readmission (*p* for nonlinearity = 0.009), with the lower level of TyG index increasing the risk of 6-month readmission.

Multivariable logistic regression models were used to explore the association between the TyG index and the incidence of 6-month readmission. Due to the lowest incidence of 6-month readmission in the second quartile, we categorized the TyG index into quartiles and used the second quartile as the reference (Table 2). In model 1, the lowest TyG quartile was associated with a higher incidence of 6-month readmission in the unadjusted model 1 (OR 1.74, 95%CI 1.18–2.57; *p*=0.005), partially adjusted model 2 (OR 1.82, 95%CI 1.22–2.72; *p*=0.004), and fully adjusted model 3 (OR 1.65, 95%CI 1.09–2.45; *p*=0.024). These results suggested that a lower TyG index independently increased the risk of 6-month readmission in HF patients.

**Figure 2:**
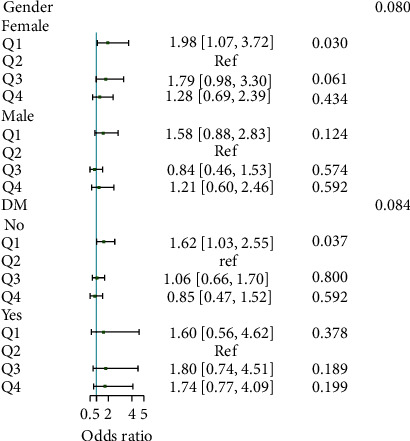
Subgroup analysis of the association between TyG quartile and 6-month readmission of HF patients.

Subgroup analysis was performed to explore the interaction factors modifying the associations between the TyG index and the incidence of 6-month readmission (Figure 2). Even though a higher risk in female or nondiabetic individuals, the interaction was not significant. The association was consistent across gender and DM group. Furthermore, we divided DM patients into well and poorly controlled blood glucose and reanalysis again (Table 3). The results also showed that the TyG index was nonlinearly associated with 6-month readmission in heart failure patients and a lower level of TyG increased the risk of 6-month readmission. While in DM patients with well controlled blood glucose (<7 mmol/L), a relative higher TyG index was associated with the highest risk of 6-month readmission. However, the number of DM patients with well controlled blood glucose was small and the conclusion was needed to confirm in a large cohort.

## 4. Discussion

In this study, we found that the TyG index was nonlinearly associated with 6-month readmission in heart failure patients. Specially, a lower TyG index (<7.36) independently increased the risk of 6-month readmission in HF patients. These results confirmed that TyG index could be a reference value and a predictor in HF patients.

Identifying patients with heart failure who are most at risk of readmission is meaningful for targeting interventions. A real-world study found that readmission of HF occurred in early postdischarge period and was driven by worsening HF. The common risk factors include co-morbidity and poor compliance to medications [13]. Based on the serum levels of glucose and triglyceride, we identified that the TyG index was a predictor of 6-month readmission in HF patients. The elevated TyG index is a predictive factor of adverse cardiovascular events in myocardial infarction with nonobstructive coronary arteries [14], coronary artery disease [8], hypertension [7], and chronic heart failure [9].

A dose-response meta-analysis showed that the relationship between TyG index and major adverse cardiovascular events was nonlinear, and a TyG index above 8.9 predicted the poor prognosis [15]. Our study also confirmed a nonlinear association between the TyG index and 6-month readmission with a TyG index below 7.36 increasing the risk of readmission. The association was independent on other risk factors, including gender and diabetes. The participants with the higher quartiles have more presence of DM, CKD or other confounding factors, which could increase the risk of readmission of HF and weaken the association of TyG. Besides, a higher TyG index may be related to a good nutrition status and be compensatory for the development of HF.

The potential mechanisms underlying the association between the TyG index and the risk of readmission in HF patients have not been fully elucidated. First, insulin resistance is an adaptive mechanism that prioritizes utilization of energy and may be protective for HF readmission [16]. Secondl, the TyG index was a marker of cardiac fibrosis [10], which may have an effect on impaired cardiac function.

There are several limitations in the present study. First, the sample size was small to assess the relationship of the TyG index with readmissions of heart failure. Second, the types of heart failure were not distinguished, for example, heart failure with preserved EF or reduced EF. Finally, the TyG index was collected once on admission, therefore, the effect of longitudinal changes in the TyG index on the risk of readmissions in HF patients remains uncertain. It may be better to monitor lipid panel more frequently in HF patients.

## 5. Conclusions

TyG index was nonlinearly associated with 6-month readmission in heart failure patients. Specially, a lower TyG index independently increased the risk of 6-month readmission in HF patients. This makes a strong case for target ranges for triglycerides and glucose rather than target levels.

## Figures and Tables

**Table 1:** The baseline characteristics of HF patients according to the TyG quartile.

	Q1 (*n* = 224)	Q2 (*n* = 228)	Q3 (*n* = 225)	Q4 (*n* = 224)	*p* value
*Age, yrs*					0.152
<60	13 (5.8)	15 (6.6)	15 (6.7)	25 (11.2)	
60∼80	112 (50.0)	107 (46.9)	122 (54.2)	115 (51.3)	
>80	99 (44.2)	106 (46.5)	88 (39.1)	84 (37.5)	
Male, %	108 (48.2)	115 (50.4)	104 (46.2)	74 (33.0)	0.001
sBP, mmHg	128.9 ± 23.9	133.2 ± 25.5	132.7 ± 23.5	134.8 ± 28.2	0.09
dBP, mmHg	75.0 ± 13.1	78.1 ± 13.8	77.3 ± 14.1	77.8 ± 17.2	0.103
BMI, kg/m^2^	20.9 ± 3.8	21.3 ± 3.5	21.4 ± 4.2	21.8 ± 4.6	0.142
*NYHA, %*					0.965
II	37 (16.5)	37 (16.2)	35 (15.6)	36 (16.1)	
III	106 (47.3)	105 (46.1)	115 (51.1)	110 (49.1)	
IV	81 (36.2)	86 (37.7)	75 (33.3)	78 (34.8)	
MI, %	16 (7.1)	14 (6.1)	22 (9.8)	22 (9.8)	0.371
CHF, %	206 (92.0)	218 (95.6)	206 (91.6)	208 (92.9)	0.322
Hypertension, %	69 (30.8)	89 (39.0)	81 (36.0)	90 (40.2)	0.165
PAD, %	11 (4.9)	21 (9.2)	12 (5.3)	11 (4.9)	0.159
DM, %	29 (12.9)	41 (18.0)	50 (22.2)	112 (50.0)	<0.001
CKD, %	48 (21.4)	55 (24.1)	48 (21.3)	68 (30.4)	0.088
LVEF, %	53.2 ± 12.9	49.6 ± 13.8	48.1 ± 13.1	52.1 ± 13.2	<0.001
LVEDD, mm	53.6 ± 11.1	53.2 ± 10.6	54.9 ± 9.6	51.2 ± 9.8	0.002
HsCRP, mg/L	23.8 ± 34.4	26.7 ± 34.5	20.7 ± 31.3	29.6 ± 40.5	0.053
BNP, pg/mL	1260 ± 1312	1534 ± 1488	1496 ± 1517	1234 ± 1380	0.045
Albumin, g/L	35.32 ± 4.53	35.82 ± 4.70	36.61 ± 4.73	36.86 ± 5.31	0.002
eGFR, mL/min/1.73 m^2^	76.4 ± 41.6	69.3 ± 35.7	66.9 ± 31.4	66.2 ± 38.2	0.014
Cholesterol, mmol/L	3.22 ± 0.92	3.63 ± 0.93	3.87 ± 0.97	4.37 ± 1.36	<0.001
LDL, mmol/L	1.47 ± 0.60	1.81 ± 0.65	1.99 ± 0.69	2.25 ± 0.95	<0.001
HDL, mmol/L	1.16 ± 0.40	1.11 ± 0.36	1.14 ± 0.35	1.08 ± 0.33	0.079
Triglyceride, mmol/L	0.63 ± 0.16	0.87 ± 0.21	1.07 ± 0.26	1.97 ± 1.75	<0.001
Glucose, mmol/L	5.48 ± 1.38	6.54 ± 1.69	7.89 ± 2.32	11.64 ± 5.97	<0.001
TyG index	7.03 ± 0.25	7.53 ± 0.10	7.91 ± 0.13	8.73 ± 0.55	<0.001
ACEI/ARB	168 (75.0)	181 (79.4)	169 (75.1)	170 (75.9)	0.428
MRA	190 (84.8)	192 (84.2)	191 (84.9)	189 (84.4)	0.634
Death_1M	5 (2.2)	5 (2.2)	5 (2.2)	9 (4.0)	0.55
Readmission_1M	18 (8.0)	14 (6.1)	12 (5.3)	17 (7.6)	0.64
Death_3M	5 (2.2)	6 (2.6)	5 (2.2)	9 (4.0)	0.615
Readmission_3M	54 (24.1)	46 (20.2)	39 (17.3)	52 (23.2)	0.28
Death_6M	6 (2.7)	11 (4.8)	6 (2.7)	10 (4.5)	0.47
Readmission_6M	94 (42.0)	67 (29.4)	74 (32.9)	75 (33.5)	0.036

TyG, triglyceride glucose. Q1: 6.17∼7.36, Q2: 7.37 ∼7.70, Q3: 7.71∼8.13, Q4: 8.14∼11.86. sBP, systolic blood pressure; dBP, diastolic blood pressure; BMI, body mass index; NYHA, New York Heart Association; MI, myocardial infarction; CHF, chronic heart failure; PAD, peripheral artery disease; DM, diabetes mellitus; CKD, chronic kidney disease; LVEF, left ventricular ejection fraction; LVEDD, left ventricular end diastolic diameter; hsCRP, high sensitivity C-reactive protein; BNP, brain natriuretic peptide; eGFR, estimated glomerular filtration rate; LDL, low-density lipoprotein cholesterol; HDL, high-density lipoprotein cholesterol; ACEI/ARB, angiotensin converting enzyme inhibitors/angiotensin receptor blockers; MRA, mineralocorticoid receptor antagonists.

**Table 2:** The association between TyG quartile and 6-month readmission of HF patients.

	*Model 1*		*Model 2*		*Model 3*	
	OR (95%CI)	*p*	OR (95%CI)	*p*	OR (95%CI)	*p*
Q1	1.74 [1.18, 2.57]	0.005	1.82 [1.22, 2.72]	0.004	1.65 [1.09, 2.45]	0.024
Q2	Ref	-	Ref	-	Ref	-
Q3	1.18 [0.79, 1.76]	0.421	1.20 [0.79, 1.81]	0.396	1.18 [0.71, 1.80]	0.468
Q4	1.21 [0.81, 1.80]	0.348	1.13 [0.74, 1.74]	0.573	1.12 [0.72, 1.82]	0.588

Model 1: unadjusted; Model 2: adjusted for age, gender, BMI, NYHA, MI, CHF, hypertension, PAD, COPD, DM, CKD, LVEF, and LVEDD; Model 3: adjusted for age, gender, BMI, NYHA, MI, CHF, hypertension, PAD, COPD, DM, CKD, LVEF, LVEDD, ACEI/ARB, MRA, eGFR, hsCRP, BNP, albumin, cholesterol, and LDL. OR, odds ratio; CI confidence interval.

**Table 3:** The association between TyG quartile and 6-month readmission of HF patients in DM patients with well and poorly controlled blood glucose.

DM patients	HR (95%CI)	*p* for interaction
Glucose ≤7 (*n* = 76)		0.045
Q1	3.15 [0.41, 28.40]	
Q2	Ref	
Q3	31.61 [3.21, 528.05]^*∗∗*^	
Q4	0.75 [0.03, 14.87]	

Glucose >7 (*n* = 156)		
Q1	3.45 [0.19, 110.24]	
Q2	Ref	
Q3	0.81 [0.20, 3.41]	
Q4	0.82 [0.22, 3.23]	

OR, odds ratio; CI confidence interval.

## Data Availability

The dataset is available at PhysioNet.
